# The role of artificial intelligence in coronary CT angiography

**DOI:** 10.1007/s12471-024-01901-8

**Published:** 2024-10-10

**Authors:** Rudolf L. M. van Herten, Ioannis Lagogiannis, Tim Leiner, Ivana Išgum

**Affiliations:** 1 Department of Biomedical Engineering and Physics, Amsterdam University Medical Center—location University of Amsterdam, Amsterdam, The Netherlands; 2 Informatics Institute, University of Amsterdam, Amsterdam, The Netherlands; 3 Amsterdam Cardiovascular Sciences, Amsterdam University Medical Center—location University of Amsterdam, Amsterdam, The Netherlands; 4 Department of Radiology, Mayo Clinic, Rochester, MN USA; 5 Department of Radiology, University Medical Center Utrecht, Utrecht, The Netherlands; 6 Department of Radiology and Nuclear Medicine, Amsterdam University Medical Center—location University of Amsterdam, Amsterdam, The Netherlands

**Keywords:** Coronary artery disease, Coronary CT angiography, Artificial intelligence, Deep learning

## Abstract

Coronary CT angiography (CCTA) offers an efficient and reliable tool for the non-invasive assessment of suspected coronary artery disease through the analysis of coronary artery plaque and stenosis. However, the detailed manual analysis of CCTA is a burdensome task requiring highly skilled experts. Recent advances in artificial intelligence (AI) have made significant progress toward a more comprehensive automated analysis of CCTA images, offering potential improvements in terms of speed, performance and scalability. This work offers an overview of the recent developments of AI in CCTA. We cover methodological advances for coronary artery tree and whole heart analysis, and provide an overview of AI techniques that have shown to be valuable for the analysis of cardiac anatomy and pathology in CCTA. Finally, we provide a general discussion regarding current challenges and limitations, and discuss prospects for future research.

## Introduction

**Fig. 1:**
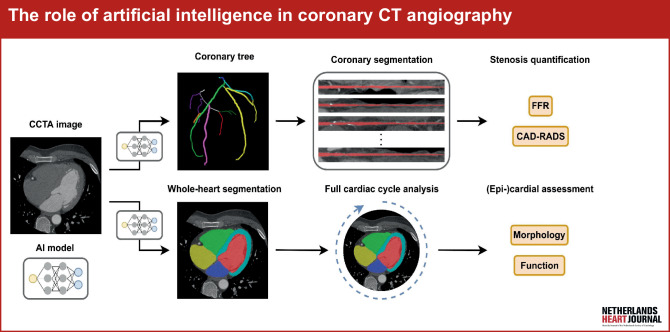
Infographic: Flowchart for AI-driven analysis of coronary CT angiography

Coronary artery disease (CAD) remains the most common cause of death globally [1]. With the recent introduction of multidetector row CT scanners capable of short rotation times, capturing a high spatial resolution image of the heart and coronary arteries in one to several heartbeats is now possible. Because of this, coronary CT angiography (CCTA) offers unparalleled opportunities to reduce the burden of disease by accurate detection of coronary calcium as well as coronary plaque. Advanced modelling and image analysis also allow for probing the haemodynamic significance of coronary artery stenoses and, as a result, CCTA recently received a class 1, evidence level A indication for use as a first-line tool to evaluate patients with suspected CAD [1]. However, detailed analysis of CCTA remains labour intensive and demands highly skilled experts for image interpretation, which currently limits access [2]. Recent advances in artificial intelligence (AI) provide excellent opportunities to dramatically improve the speed and level of detail with which CCTA can be analysed [2]. Furthermore, CCTA examinations contain much more data than can be manually analysed by even the most experienced observers. Here we review recent advances in AI relevant to CCTA with a specific emphasis on deep learning developments in scientific research, focusing on both the coronary arteries and the heart as a whole (see Fig. 1). An overview of discussed methods for these applications is presented in Tab. 1.

**Table 1:** An overview of the applications discussed in this paper. For each application, the relevant cited methods are presented by type. We distinguish non-deep-learning approaches (conventional), artificial neural networks (ANN), convolutional neural networks (CNN), recurrent neural networks (RNN), graph convolutional networks (GCN), and physics-informed neural networks (PINN)

	Non-DL	ANN	CNN	RNN	GCN	PINN
Centerline extraction	[6, 11]		[7–9]		[10]	
Coronary tree labeling	[12]			[13]	[14, 15]	
Coronary plaque and anatomical stenosis assessment	[20, 25]		[16–19, 23, 24, 26, 27]	[17, 25, 26]	[21, 22]	
Functionally significant stenosis	[37]	[31–34]	[38–41]		[35]	[36]
Whole heart analysis	[43, 44]	[47]	[42, 45, 47, 49]		[46]	

## Coronary artery tree analysis

Analysis of the coronary arteries lies at the core of CCTA, as it provides direct insights into CAD [3]. Obstruction of the major blood vessels supplying the heart may lead to anatomically and functionally significant stenoses, and is characterised by various pathologies [4]. Due to the large variation in anatomy and pathology, the automated analysis of CCTA with the help of AI has gained much interest.

### Centreline extraction

Coronary artery centreline extraction is a prerequisite for manual and automatic CCTA analysis. Extracted centrelines enable the generation of multi-planar reconstruction (MPR) and curved multi-planar reconstruction (cMPR) images, which are routinely used during CCTA-based CAD diagnosis [5]. Furthermore, automatic CCTA analysis frameworks rely on coronary artery centrelines and their corresponding MPRs. Since manual extraction is labour intensive, numerous automatic and semi-automatic methods for coronary artery centreline extraction have been proposed [6–10].

**Fig. 2:**
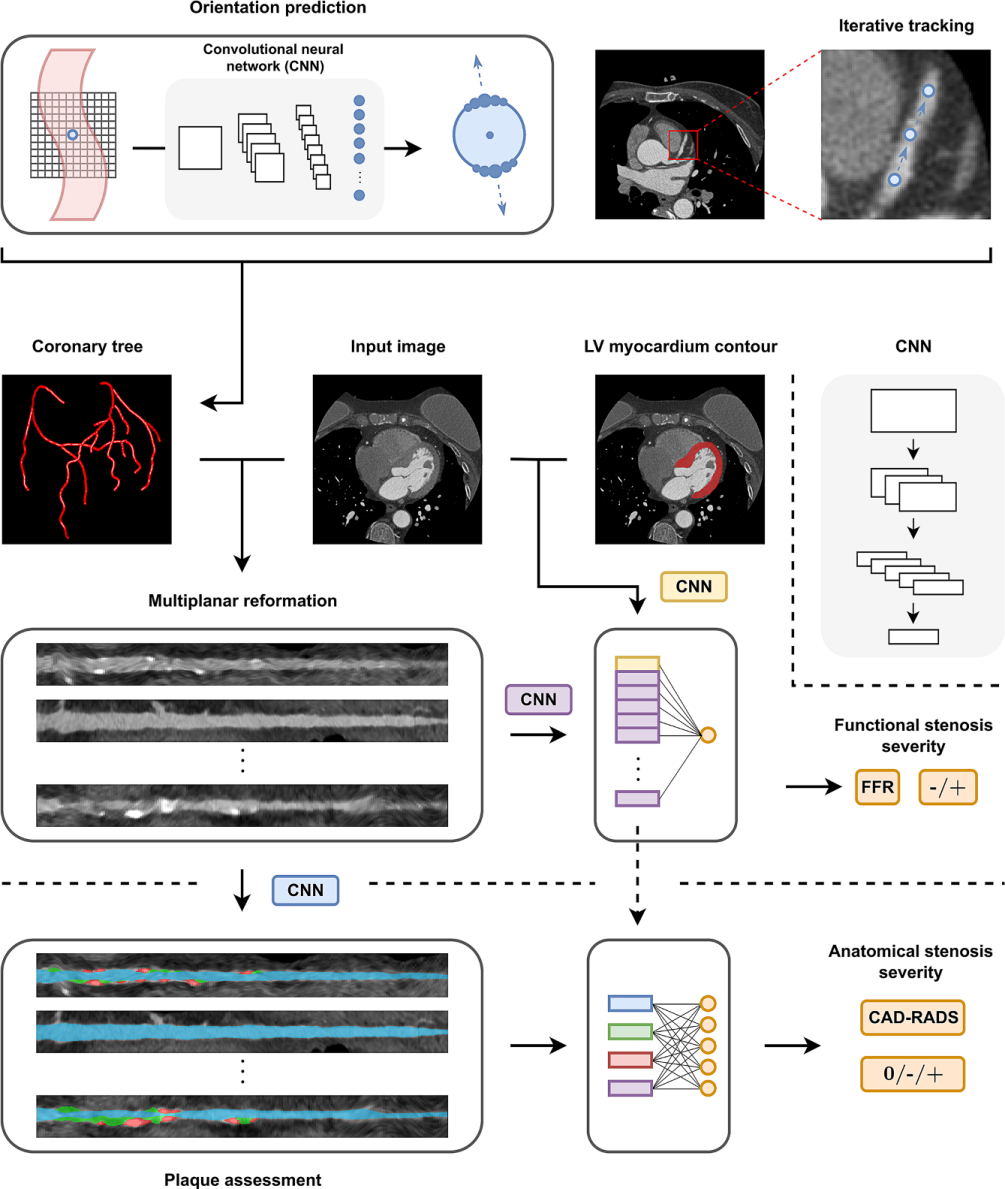
Workflow of the image-based analysis of coronary arteries to assess anatomical and functional stenosis severity. Top: coronary artery centrelines are first extracted automatically by iteratively predicting the vessel orientation for image patches centred on points along the coronary artery [7]. Multiplanar reformatted (MPR) images are subsequently extracted from centrelines. Middle: A neural network extracts features from both MPRs and the left ventricle myocardium. Features are used to predict the severity and/or presence of any functional stenoses [39]. Bottom: A lumen and plaque segmentation step is performed, which can subsequently be used to predict the severity of anatomical stenosis

In conventional approaches, centrelines were extracted by connecting two semi-automatically defined vessel points in a CCTA volume, using traditional minimum cost path techniques [11], or trackers [6], often relying heavily on user interaction. Wolterink et al. [7] were the first to use convolutional neural networks (CNNs) instead of hand-crafted filters to identify coronary artery seed points and guide a tracker without requiring any manual interaction (Fig. 2). Nevertheless, tracking through severe cases of stenosis and calcifications or extremely tortuous vessels remained challenging. Hence, subsequently developed approaches focused on mitigating this issue [8, 9]. Recently, Alblas et al. [10] designed a tracker that exploits rotation and scale symmetries inherent to vascular data, allowing it to generalise well to various degrees of scale and tortuosity. Notably, their method accurately tracked abdominal aorta centrelines when trained exclusively on coronary artery data, thus demonstrating generalisability to anatomies unseen during training.

### Coronary tree labelling

According to the guidelines, CCTA reports should include segment-level information on the location of detected atherosclerotic lesions and stenosis [5]. Automatic labelling of the coronary tree could assist physicians by streamlining the diagnostic workflow.

Atlas-based registration [12] was initially investigated, but was limited in its ability to handle the morphological variability of the coronary tree. Recently, deep neural networks (DNNs) have shown to be better suited for the task, as their expressive power allows them to handle large inter-patient anatomical variations more effectively. Motivated by its innate structure, deep-learning researchers typically represent the coronary tree as a graph, whose nodes correspond to coronary segments. This approach can be traced back to the first DNN-based segment labelling framework proposed by Wu et al. [13], who used recurrent neural networks (RNNs) to model the graph representations of coronary trees. Recent research has suggested using graph neural networks (GNNs) to leverage the inherent graph structure. Yang et. al [14] were among the first to leverage GNNs for segment labelling. Their method outperformed the RNN-based approach and was especially robust against corrupted coronary tree data. Recently, Zhang et al. [15] recognised the potential of using the predetermined interconnections of coronary segments as prior knowledge. By injecting strict topological priors into a GNN-based architecture, they enforced anatomically plausible labelling and set the new state-of-the-art in the field.

### Coronary plaque and anatomical stenosis assessment

Identifying and reporting the location and severity of anatomical stenosis is the primary goal of clinical CCTA interpretation in the context of CAD diagnosis [5]. However, visual assessment can be a challenging and labour-intensive task susceptible to inter-observer variability. Therefore, an automatic stenosis assessment framework may improve the efficiency and reliability of the diagnostic workflow.

Automatic stenosis assessment typically relies on an initial lumen segmentation step, followed by stenosis localisation and estimation of its degree by direct stenosis measurement [16, 17], or post-processing the segmented volumes [18, 19]. Although early works on lumen segmentation employed traditional computer vision techniques [20], more recent works have favoured deep learning approaches [19–22]. For example, Hong et al. [16] showed that stenosis biomarkers detected from DNN-derived lumen segmentations correlated strongly with expertly defined stenosis measurements. Li et al. [18] proposed to segment the coronary tree first, followed by a DNN to detect significant stenosis. While these works adopted a conventional voxel-based approach, voxel-wise segmentation might fail to meet the quality demands of downstream tasks. To that end, alternatives to the voxel-based paradigm have gained traction, in which researchers leverage deformable shape priors that can achieve sub-voxel accuracy and anatomically plausible, contiguous results [21, 22].

To standardise CCTA-based CAD reporting, the Coronary Artery Disease Reporting and Data System (CAD-RADS) was introduced [4], which describes a stenosis severity classification system based on the extent of anatomical stenosis in coronary arteries. Researchers have therefore proposed deep learning-based stenosis assessment methods that extract CAD-RADS scores or perform significant stenosis prediction directly from the image data. For example, Muscogiuri et al. [23] designed a CNN-based classification method demonstrating high diagnostic accuracy. Their results were surpassed by a framework proposed by Denzinger et al. [24], who trained CNNs to predict CAD-RADS scores along with the auxiliary tasks of segment-level significant stenosis and patient-level calcium score prediction, showing that combining CAD-RADS prediction with highly correlated secondary targets can offer substantial benefits to model performance.

**Fig. 3:**
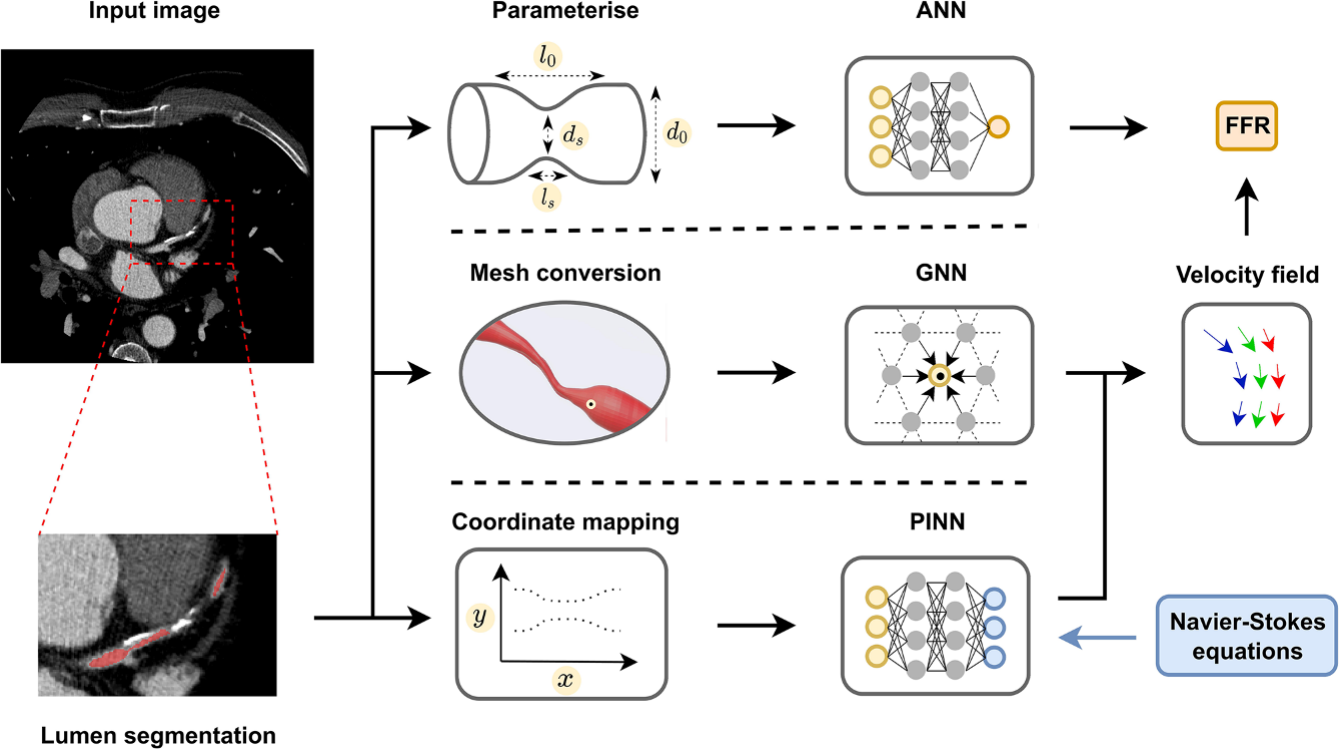
Geometry-based surrogates for assessment of functional stenosis severity. One option is the parametrisation of a stenotic lesion in terms of geometric features, as proposed by Itu [32]. Features are used to train an artificial neural network (ANN) to directly predict fractional flow reserve. A neural network may also directly operate on the automatically extracted luminal mesh by using graph neural networks [36]. These may be used to predict velocity fields along the coronary artery. Finally, velocity fields may be identified by learning a physics-informed neural network (PINN) [37] to fit the Navier-Stokes equations along with the artery geometry

Plaque-type characterisation to either calcified, non-calcified, or mixed is recommended during CCTA reporting [4]. Commonly, predictive models have been driven by radiomics approaches [25]. This potential of predictive models has prompted researchers to include plaque classification and quantification aspects in deep-learning-based stenosis assessment frameworks (Fig. 3). In the work by Zreik et al. [26], input MPRs were partitioned into equal volume segments and subsequently fed into an RNN that performed segment-level plaque type and stenosis severity classification. Voxel-level quantification of calcified plaque was investigated by multiple works, typically resulting in a strong correlation with coronary artery calcium scores derived from the Society of Cardiovascular Computed Tomography (SCCT) reference standard [27]. Lin et al. [15] performed voxel-level plaque characterisation using RNNs for semantic segmentation of lumen, calcified and non-calcified plaque, enabling plaque burden quantification and CAD-RADS score assignment based on stenosis measurement. In a multi-cohort validation, deep learning-based plaque burden measurements were shown to correlate excellently with expert visual assessment and intravascular ultrasound, and stenosis measurements correlated strongly with intravascular coronary angiography. DNN-derived total plaque volumes demonstrated predictive value for future cardiac events. Recently, a deep learning-based pipeline was proposed by Van Herten et al. [19]. Here, cylindrical shape priors are leveraged to extract surface meshes for the lumen, calcified and non-calcified plaque using a 3D CNN. The meshes are then reformatted to one-dimensional signals and passed as inputs to a CNN that performs CAD-RADS score prediction.

### Functionally significant stenosis

The functional significance of stenosis is determined by the degree to which stenosis limits the downstream blood flow of a coronary artery [28]. In practice, this is measured invasively by the fractional flow reserve (FFR), which calculates the pressure ratio between the regions distal and proximal to a stenotic lesion [29]. Since this invasive measurement is costly and burdensome for patients, the non-invasive quantification of functional stenosis severity from CCTA has gained much interest [30]. Several algorithms have been proposed for the automated prediction of FFR from CCTA, which can generally be subdivided into two categories.

**Fig. 4:**
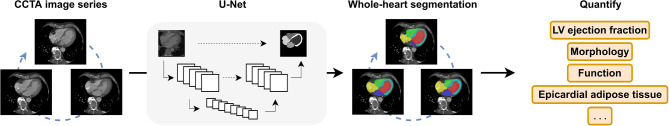
Whole heart image analysis typically involves the segmentation of cardiac structures such as the cardiac chambers and the epicardium. Given a CCTA image series over the cardiac cycle, this allows for the quantification of valuable clinical risk predictors such as the left ventricle ejection fraction, cardiac morphology and function, and the presence of epicardial adipose tissue

The first category of algorithms considers geometric and anatomical coronary artery tree features for the non-invasive analysis of coronary artery flow from CCTA [31–36]. In the conventional computational fluid dynamics (CFD) approach, (voxelwise) lumen segmentations are first converted into triangulated meshes, which allows finite-element methods to estimate blood flow parameters by iteratively solving differential equations [37]. Since such methods are computationally expensive, recent years have seen a surge in research focussing on surrogate models for efficient blood flow parameterisation. The fast inference of DNNs has made them a popular choice for such a surrogate model; an overview is presented in Fig. 4.

For example, Itu et al. [31] developed an artificial neural network (ANN) to estimate FFR at each point along the coronary artery based on anatomical and stenosis severity features. They trained the network using 12,000 simulated coronary tree anatomies with reference FFR values from CFD simulations. The proposed ANN reduces computation time by more than 80 times compared with conventional CFD-based approaches, and has been evaluated extensively [32]. Yu et al. [33] further expanded on the work by Itu et al. [31] by providing the ANN with the CT morphological index, increasing its predictive value. Long-range dependencies between arterial segments were later researched by modelling FFR values through recurrent ANNs [34].

Alternatively, deep learning methods can infer complete 3D velocity fields, which enables direct calculation of FFR. Suk et al. [35] used GNNs, which directly operate on input mesh geometries to predict velocity fields. As in Itu et al. [31], the method was trained on generated coronary tree anatomies for which reference velocity fields were calculated through CFD. The GNN can predict near-instant velocity fields at inference given only a small amount of training data.

Deep learning has further been leveraged to solve the underlying differential equations in CFD directly. Specifically, Raissi et al. [36] proposed physics-informed neural networks (PINN) to predict dense velocity fields by implicitly solving the Navier-Stokes equation given the arterial wall boundary conditions. This offers several advantages over conventional CFD approaches, such as robustness to low resolution and noisy observed data. However, this method is relatively slow compared with other deep learning alternatives due to its iterative nature, similar to CFD.

A downside to deep learning-based CFD surrogate models is that they rely on lumen segmentations to correctly predict functional stenosis, for which an accurate reconstruction of the arterial geometry is essential. Therefore, the second category of algorithms focuses on directly predicting functionally significant stenosis from image data rather than a geometric data-based approach [38–41]. This is typically achieved by training a computer vision model on regions of interest in CCTA images.

The coronary artery tree, and in some cases the left ventricular myocardium, are considered regions of interest for the analyses. These are identified through a pre-processing segmentation step. For example, Zreik et al. [38] proposed a CNN to extract features from the myocardium, after which a support vector machine classifies patient-level functional stenosis severity. Later work expanded on this by performing a combined CNN-based analysis of the coronary artery tree and the myocardium, further improving the diagnostic accuracy of functional stenosis prediction [39]. Hampe et al. [40] proposed an explainable method, in which the authors characterised coronary arteries in terms of anatomical and pathological information obtained from multi-planar reformatted images and the coronary artery tree. A small neural network subsequently directly regressed the FFR value, and classified functional significance of the stenosis. A different approach not requiring segmentation was proposed by Kumamaru et al. [41]. The method performs end-to-end detection of functional stenosis and regression of FFR by automatically identifying abnormal regions surrounding the coronary artery lumen.

## Whole heart analysis

Analysis of cardiac chambers and large arteries may provide valuable insights into cardiovascular risk prediction. Since manual cardiac delineation takes approximately 5 h per 3D image volume [42], this analysis is not routinely performed in clinical settings. However, deep learning approaches can help identify and quantify key structural features.

### Whole heart segmentation

Many cardiac analyses such as cardiac output quantification, strain imaging and epicardial tissue segmentation require delineating structures of interest. In the case of whole heart analysis, this typically involves analysis of the cardiac chambers, the left ventricular myocardium and the pericardium. While early methods propose model- or atlas-based segmentation [43, 44], deep learning-based methods have been shown to outperform these traditional approaches. Most deep learning-based methods consider the voxelwise segmentation of cardiac structures using CNNs, in which each voxel is categorised into a specific class that represents the cardiac structure it belongs to (Fig. 4). Such methods result in highly precise delineations [42, 45].

A downside to voxelwise segmentation is that this approach typically does not penalise anatomically implausible shapes or nonphysical artifacts. Some studies therefore leverage cardiac shape constraints for segmentation. For example, Kong et al. [46] proposed to reconstruct surface meshes for all objects in whole heart segmentation. This was achieved by iteratively deforming a sphere to match the cardiac shape of interest with a GNN, resulting in a high-resolution cardiac mesh. Attar et al. [47] proposed combining image data with patient metadata to extract a feature representation. These are then used to construct a mesh representation of the cardiac chambers.

### Downstream analysis

To perform a functional analysis of the whole heart, a growing number of clinical studies acquire 3D CCTA scans over the full cardiac cycle. Given segmentations of these scans, downstream analyses may be performed to quantify cardiac function. For example, Bruns et al. [42] conducted a morphological analysis of the treatment planning images acquired for transcatheter aortic valve implantation, in which the authors demonstrated accurate quantification of the left ventricular ejection fraction. Szilveszter et al. [48] showed the feasibility of left ventricular and atrial strain imaging using CCTA by tracking deformation vectors. Furthermore, delineations of the pericardial boundary were leveraged to quantify epicardial adipose tissue by Commandeur et al. [49]. Epicardial and perivascular adipose tissue deposits have been shown to impact cardiovascular risk assessment in recent years [49].

## Discussion

In this work, we have presented the role of AI in recent research concerning the analysis of CCTA images. Specifically, we covered AI techniques that have been leveraged for relevant clinical tasks in CCTA, and aspects that these new methodologies offer to analyse the coronary artery tree and whole heart. These developments provide insights into the current state of research, opportunities and limitations for CCTA analysis.

An analysis of automatic coronary artery centreline tracking shows that most methods are framed as orientation classifiers, which predict the direction of a coronary artery from a local image patch. Though these methods perform well and can be applied to arteries of different scales [10], their local nature makes it nearly impossible to trace beyond total occlusions. Since this is an important clinical categorisation, it would be interesting for centreline trackers to incorporate stenosis awareness or grading. This may also improve downstream analyses, which rely heavily on correct centreline extraction.

The automated analysis of functionally significant stenoses especially has seen several technological advancements for both image- and CFD surrogate-based methods. Deep learning methods can for example be directly regulated through differential equations [36], or be constrained by step-by-step approaches using intermediate results such as lumen area regression [40]. However, methods have not yet matched the performance of invasive measurements, which could be due to several reasons. For example, FFR has been shown to vary substantially for slight variations in points distal and proximal to suspected culprit lesions. Furthermore, image analysis of MPR volumes may limit the prediction of functional stenosis, as it does not account for the effects of coronary artery curvature and torsion.

A general remark can be made regarding the trustworthiness of recently introduced AI methods. Due to variations in image quality, dataset size, and ground truth availability, it is often difficult to compare proposed methods. Furthermore, false-positive and false-negative outputs may be difficult to explain due to the black-box nature of most AI systems. Therefore, proposed pipelines have incorporated explainability to some degree, ranging from intermediate segmentation steps for anatomical stenosis prediction [17, 19] to velocity field prediction [35] which may be used in downstream functional analyses. Additionally, image quality may be improved through advancements in CT reconstruction, noise reduction and motion artifact reduction. Such methods can aid in the robustness of AI systems and provide opportunities for further improvements.

### Outlook

Although all presented works contribute to the automated analysis of CAD from CCTA, a lack of standardisation in data availability and evaluation creates challenges in objectively comparing performances between methods. Works differ vastly in training and testing data, and the distribution of pathologies within those sets. Moreover, aspects of trustworthy AI are typically not evaluated. Although evaluations on open datasets may alleviate these issues to some extent, the size, availability and evaluation criteria of such sets are typically limited [10, 45]. An initiative towards a large, multi-centre, curated dataset including clinical outcomes could benefit the development, validation, and eventual clinical adaptation of models.

The recent emergence of foundation models may further change the scene of CCTA segmentation algorithms, as the effective finetuning of such models may yield a high performance even with few annotated examples.

An interesting future development involves the analysis of both photon-counting and spectral CT, which provide significant improvements in image quality and diagnostic accuracy over traditional CT [50]. Photon-counting CT, in particular, is expected to enhance automated CAD analysis in the coming years.

In conclusion, we have provided an overview of recent developments utilising AI to analyse CCTA images for the automated diagnosis of CAD. We highlight the steps and methods introduced for coronary artery and whole heart analysis.
